# Spanish Cross-Cultural Adaptation of the Wisconsin Gait Scale

**DOI:** 10.3390/ijerph18136903

**Published:** 2021-06-27

**Authors:** Cecilia Estrada-Barranco, Vanesa Abuín-Porras, Javier López-Ruiz, Ismael Sanz-Esteban, Francisco Molina-Rueda, Roberto Cano-de-la-Cuerda

**Affiliations:** 1 Department of Physiotherapy, Faculty of Sport Sciences, Universidad Europea de Madrid, Villaviciosa de Odón, 28670 Madrid, Spain; Cecilia.estrada@universidadeuropea.es (C.E.-B.); vanesa.abuin@universidadeuropea.es (V.A.-P.); Javier.Lopez3@universidadeuropea.es (J.L.-R.); Ismael.sanz@universidadeuropea.es (I.S.-E.); 2 Department of Physical Therapy, Occupational Therapy, Physical Medicine and Rehabilitation, Faculty of Health Sciences, Rey Juan Carlos University, Alcorcón, 28922 Madrid, Spain; roberto.cano@urjc.es

**Keywords:** biomechanics, gait, postural balance, stroke, patient outcome assessment

## Abstract

Introduction: the Wisconsin Gait Scale (WGS) has been shown to be a valid and quick tool for analyzing gait in post-stroke people in the clinical setting. Its widespread use has led to versions of the scale in other languages, but so far there has been no version in Spanish. Objective: to conduct a cross-cultural adaptation of the WGS for use in the Spanish-speaking population and to analyze the content validity. Materials and methods: the Spanish version was obtained using the double translation method and back translation method, followed by a review by an expert committee. This expert committee evaluated the content validity index (CVI) for each item on the version obtained and for the entire scale (scale content validity index (S-CVI)). The item content validity index (I-CIV) was calculated as the number of experts whose score had been 3 or 4 divided by the total number of experts. To obtain the S-CVI, the middle of the I-CVI was calculated for all the items on the scale. Results: the Spanish version of the WGS was obtained after the expert committee evaluation. The CVI was excellent for its general assessment (0.91), excellent for 85.7% of its items (≥0.78), and good for 14.3% of the CVI (≥0.72). Conclusions: the Spanish version of the WGS was developed through a process of cross-cultural adaptation from its original English version, and, according to an expert committee, it had an excellent content validity.

## 1. Introduction

Gait analysis is an essential part of the evaluation of a patient who has suffered a stroke. Understanding the problems that have a negative effect on the gait pattern provides information on the patient’s functional status, helps with the design of therapeutic strategies, and quantifies the progress achieved through rehabilitation [1]. Several walking alterations have been described in stroke patients: pelvic anteversion during the stance period, increased or decreased hip and knee flexion during the initial contact, and toe-off and increased ankle plantar flexion during the toe-off [2].

Three-dimensional motion capture analysis provides objective data on electromyography and kinematic, kinetic, and spatiotemporal parameters [3]. However, because of its high cost in the clinical setting, the use of observational tests and assessment scales that quantify and evaluate deviations from normal gait patterns is common [1]. The Wisconsin Gait Scale (WGS) has been shown to be valid for gait analysis in patients who have suffered a stroke [4,5,6,7]. It has also shown an excellent correlation with three-dimensional analysis systems [8] and with scales of balance, functionality, and independence for gait at different periods of evolution after the stroke [9].

The WGS was developed in 1996 by Rodriquez et al. [10] to analyze the progress of subjects with hemiparesis in the field of patient rehabilitation. It is comprised of 14 items that add up to a maximum score of 42 points and a minimum score of 13.35. Therefore, the lower the score, the higher the quality of the gait pattern [6,10]. The scale evaluates the kinematics of the hip, knee, and ankle during the stance and swing phases of the gait, as well as the symmetry between the two sides of the body, the balance, or the need for technical aids and other relevant parameters in the assessment of gait, such as the length of step or the duration of the stance phase on the most-affected side [5].

The WGS was originally developed in English. However, its use is widespread in many countries, which underlines the need to adapt it for use in other nations or population groups with other languages [11]. Therefore, a simple translation is not enough, as it must include cultural differences between the original target population and the new target population. This process is known as cross-cultural adaptation and content validity analysis [11]. The content validity of a scale refers to its ability to use all its items to assess the construct that it claims to measure [12]. This is assessed by a committee of experts who analyze whether the points are important, representing the concept to be measure [13]. To our knowledge, there is no version of the WGS officially translated into Spanish.

Therefore, this work set out to perform a cross-cultural adaptation of the WGS into Spanish and aimed to study the content validity of the generated version.

## 2. Materials and Methods

### 2.1. Cross-Cultural Adaptation Process

To perform the transcultural adaptation of the WGS, we followed the guidance of Beaton, Bombardier, Guillemin, and Ferraz [11], carrying out the process below:

- Direct translation: in keeping with this guidance, we contracted two translators (T1 and T2) to perform separate translations of the original scale. Neither translator knew the scale beforehand. One of them was a physiotherapist, who was able to better understand the clinical setting of the scale, and the other was a “naïve translator,” who did not know about the content of the scale or its clinical significance. They were bilingual in English and Spanish.
- Synthesis: both translations, T1 and T2, were compared and synthesized by the two translators, with the mediation of the principal investigator, resulting in the T1+2 version. Discrepancies were resolved by consensus, in the event of words without literal translation or with a literal translation that did not mean the same in Spanish.
- Back translation: two different translators from the direct translators (BT1 and BT2) then performed two independent back translations. They were bilingual and were rehabilitation experts. The idea of these back translations was to ensure the correlation between the original scale and the translation, allowing for the clarification of any word or phrase in said translations.
- Expert committee: the authors contacted by email an expert committee consisting of eight members, one of whom was a physician specializing in physical medicine and rehabilitation, and seven of whom were physical therapists. All of them had more than ten years of experience in the field of post-stroke neurological rehabilitation. The members of the expert committee reviewed the two translations (T1+2) and the two back translations (BT1+BT2) to obtain the final version of the scale.

The cross-cultural process does not completely guarantee the preservation of the psychometric properties of the scale. To reduce the bias that could be associated with the use of scales in several languages, the content validity of the scale in Spanish was evaluated [13].

### 2.2. Content Validity Analysis

The members of the expert committee rated each of the items in the Spanish version of the WGS according to the following Likert-type scale: 1: not important; 2: slightly important; 3: quite important; and 4: very important. The content validity index (CVI) was calculated for each item on the scale and for the entire scale (scale content validity index (S-CVI)) [12,14]. The content validity of each item (item content validity index (I-CVI)) was calculated as the number of experts whose score had been 3 or 4 divided by the total number of experts.

To calculate the S-CVI, the middle of the I-CVI was obtained for all the items on the scale. To be considered in the excellent range, the I-CVI score had to be >0.78, and the S-CVI had to be >0.90 [14]. To obtain the S-CVI, the middle of the I-CVI was calculated for all the items on the scale [14].

## 3. Results

With respect to the Spanish translations, there were three differences in terms of form but not content, which were mainly derived from the literal translation and the lack of clinical knowledge of one of the translators. First of all, “hand held gait aid” was translated as “walker” and as “manual support product” in each of the two translations, respectively. Secondly, “disabled side” was replaced by “affected side” by consensus, and, thirdly, “leg exit” by “swing phase.” Finally, the two back translations were very similar, reflecting some synonymous terms such as “oscillation” and “swing” and “contact” or “strike.”

In the review carried out by the expert committee, four discrepancies were identified, which were reviewed and corrected, ultimately providing the final version in Spanish. The first discrepancy appeared with the term “manual support product,” in item one, which, in Spanish, might seem to indicate that it was not electric, thereby altering the meaning of the item; so, the term “support product used with one hand” was agreed. Secondly, there was a discrepancy in the description of item five, which refers to “stance width,” as the term “shoe width” was specified by “up to one shoe width,” i.e., the shoe width as a reference. Thirdly, in the section on the swing phase of the affected side, in item six, there were discrepancies regarding the term “caution.” It was decided to translate it as “hesitancy,” which referred to the balance necessary prior to toe-off. Finally, it was decided to replace the term “movement” for the term “elevation” in item ten, as the movement referred to in the description is always elevation. 

All the corrections were incorporated into the final version of the scale, which is presented in Table 1.

**Figure 1:**
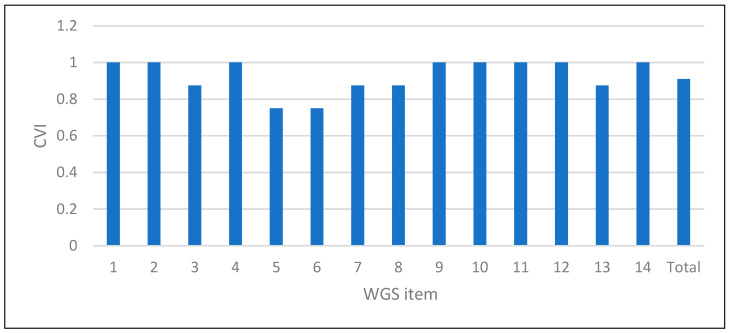
Content validity index of the WGS. CVI: content validity index.

The total content validity score (S-CVI) was 0.91, indicating excellent content validity of the WGS. Regarding the content validity by items, only 2 of the 14 items had a score below 0.78. In these two cases, the score was 0.75, which is considered a good content validity. These items were the ones that referred to “stance width prior to toe-off” and the “hesitancy” prior to the swing of the affected leg. As 12 of 14 items obtained an I-CVI > 0.78, the committee members considered them to be quite or very important (Figure 1).

## 4. Discussion

This study proposed the first cross-cultural adaptation to Spanish of the WGS, considering this an observational scale of the gait pattern. In addition, the content validity of the Spanish version of the WGS was assessed by an expert committee, showing an excellent content validity of this Spanish version. The Spanish adaptation and the content validity analysis of the WGS allows healthcare professionals to use this clinical scale in their mother tongue, avoiding errors due to the interpretation of the original language of the scale.

The cross-cultural process was specially developed for the adaptation of health-related quality of life scales [15,16,17,18]. Moreover, this methodology is adequate for the cross-cultural adaptation of any clinical measurement [19]. In this sense, most authors recommend certain steps to complete the cross-cultural adaptation process [12,15,16,19], specifically the back translation and the expert committee analysis.

Observational gait scales enable the assessment of the patient at rehabilitation centers or at their own homes in a quick, inexpensive, and accessible way [1]. The analysis of isolated gait components, such as speed or step length, has been shown not to provide enough information to guide a rehabilitation process with the objective of achieving a functional gait [20,21]. The combination of kinematic and spatiotemporal aspects has been shown to be more effective in the analysis of functional gait [1,9]. In addition, some studies have established the relationship between the gait pattern and the appearance of frequent secondary complications in stroke patients, such as osteoporosis or heart disease [20,21,22].

So far, only the G.A.I.T. scale has been adapted to Spanish [23]. Although this tool stands out among observation instruments for its level of validity, reliability, and sensitivity to changes in the gait pattern [3,23,24], it does have certain disadvantages, such as the experience required by the evaluators, the technical resources required, and the long administration time [15]. This all makes its clinical use less widespread.

The clinical significance of the WGS has led to its adaptation in various languages and countries. The WGS was adapted to Turkish by Yaliman et al. [7]. Firstly, the authors performed a double translation and back translation, but they did not include an expert committee. On the other hand, Guzic et al. adapted the WGS to Polish, using the double translation and back translation with an expert committee made up of translators, rehabilitation doctors, and physiotherapists [25]. None of these works on the WGS analyzed the content validity of the new version of the scale. 

In the present study, the content validity of the Spanish version of the WGS was high enough to justify its use. The S-CIV was 0.91, which is related to an excellent content validity [14]. As for the I-CIV, only two items had a score < 0.78, which were the two items related to the moment prior to toe-off of the most affected lower limb. In the case of item five (stance width), which assesses the distance between the feet prior to toe-off, it could be due to the fact that item five already assesses the step length, and this aspect might not be so important. In the case of item six (guardedness), this could be due to the difficulty of objectively establishing whether there is hesitancy prior to toe-off. In any case, none of these items is included in other observational gait scales such as G.A.I.T. [23], the Rivermead Visual Gait Assessment (RVGA) [26], or the Tinetti POMA [27]. However, the score of these items was 0.75, so the expert committee attributed a good content validity to them [14], and, therefore, their presence on the scale is justified.

It must be noticed that a Spanish version of the WGS has been recently published [28] but the procedure was quite different compared to ours. Firstly, three members (two physiatrists with unspecified experience and one translator) compounded their expert panel. Second, they did not analyse the CVI but rather valued the quality of the translation. For example, they only assigned each item to a category: A, if it was considered conceptually and semantically equivalent; B, when the meaning was similar, but there was some change; C, when the item presented a questionable translation, not keeping the meaning of the original item. Finally, related to the translation, they did not consider the original scale but what they called the original English version (not cited). We believe that this is an important issue when, in the original scale, the items are much more detailed than the original English version used in [28]. However, in their study, reliability, construct validity, and sensitivity were explored in stroke patients, so we believe that both investigations could be complementary.

### Study Limitations

This study presents several limitations. The guide created by Beaton and collaborators was used [15], although there were two aspects in which their recommendations were not followed. Firstly, the expert committee did not contain a language expert as such, although at least seven of the eight experts could prove that they were fluent in English as a second language. Secondly, the translators who performed the back translation did so from the Spanish version, as this was their first language. However, all translation and back translation versions were provided to the expert committee so that they could assess the process. The fact that two of the translators of the scale did not have clinical experience prevented any bias that could stem from a broad understanding of the items but increased the discrepancies of terms caused by the literal translation.

## 5. Conclusions

The version of the WGS in Spanish was developed through an intercultural adaptation process from its original English version. According to the expert committee, the WGS showed excellent content validity for its total score. The content validity was excellent for 85.7% of the items and good for 14.3%.

## Figures and Tables

**Table 1:** Spanish version of the Wisconsin Gait Scale (WGS).

Nombre		Fecha
Evaluador		Diagnóstico
Lado Evaluado		
Items	Puntuación	Descripción
Fase de Apoyo Pierna Afectada		
1. Uso de un producto de apoyo (PA) utilizado con una mano. *	1 = No usa producto de apoyo para la marcha.	
	2 = Uso mínimo de un producto de apoyo.	Uso ocasional del producto de apoyo con mínima transferencia de peso en él. Base de sustentación estrecha.
	3 = Uso mínimo de un producto de apoyo con base de apoyo amplia.	Producto de apoyo usado mínimamente. Puede apoyarse en el PA para transferir el peso hacia delante. La distancia entre el pie no afectado y el producto de apoyo es mayor que la distancia entre el pie afectado y el no afectado (base de apoyo amplia).
	4 = Uso marcado del producto de apoyo.	Peso transferido al producto de apoyo. Base de apoyo estrecha.
	5 = Uso marcado del producto de apoyo. Base de apoyo amplia.	Transfiere el peso al PA. Base de apoyo amplia.
2. Tiempo de apoyo sobre el lado afecto.	1 = Igual.	La duración de la fase de apoyo en el lado afectado es igual que la duración de la fase de apoyo en el lado sano lado no afecto, durante la fase de apoyo unipodal.
	2 = Desigual.	La duración de la fase de apoyo en el lado afectado es menor que la duración de la fase de apoyo en el lado sano, durante la fase de apoyo unipodal.
	3 = Muy breve.	El sujeto permanece apoyado sobre el lado afecto la menor cantidad de tiempo posible para lograr el avance de la pierna no afectada.
3. Longitud de paso del lado no afectado.	1 = Adelanta el pie.	El talón del pie no afectado avanza claramente más allá del primer dedo del pie afectado.
	2 = No supera el pie claramente.	El talón del pie no afectado no avanza más allá del primer dedo del pie afectado.
	3 = Paso limitado.	El pie no afectado se coloca detrás o al lado, pero no más allá del pie afectado.
4. Desplazamiento del peso hacia el lado afectado, con o sin producto de apoyo para la marcha.	1 = Desplazamiento completo.	La cabeza y el tronco del sujeto se desplazan lateralmente sobre el pie afecto durante el apoyo unipodal.
	2 = Desplazamiento reducido.	La cabeza y el tronco del sujeto cruzan la línea media pero no llegan a situarse sobre el pie afecto durante el apoyo unipodal.
	3 = Desplazamiento muy limitado.	La cabeza y el tronco del sujeto no cruzan la línea media, existe un mínimo cambio de peso en la dirección del lado afectado.
5. Ancho de paso (Mida la distancia entre los pies antes del despegue de los dedos del pie afectado).	1 = Normal.	Hasta un ancho de zapato entre pies.
	2 = Moderado.	Hasta dos anchos de zapato entre pies.
	3 = Amplio.	Más de dos anchos de zapato entre pies.
**Fase de Despegue de la Pierna Afecta**		
6. Vacilación (Existen pausas antes de avanzar la pierna afectada).	1 = Ninguna.	Buen impulso hacia delante sin vacilación.
	2 = Leve.	Pausa leve antes del despegue.
	3 = Marcada vacilación.	El sujeto se detiene antes del despegue.
7. Extensión de cadera del lado afectado. (Observe el pliegue glúteo desde detrás del sujeto.)	1 = Extensión igual.	Las caderas se extienden igual durante el empuje. Se mantiene la postura erguida durante el despegue.
	2 = Flexión leve.	Extensión neutra de cadera, pero menos que en el lado no afectado.
	3 = Flexión marcada.	El tronco se adelanta y la cadera se flexiona en el despegue del pie.
**Fase de Oscilación de la Pierna Afectada**		
8. Rotación externa durante la oscilación inicial.	1 = Igual que la pierna no afecta.	
	2 = Rotación aumentada.	Rotación externa de la pierna <45°, pero más que el lado no afecto.
	3 = Rotación marcada.	Rotación externa de la pierna >45°.
9. Circunducción en la oscilación media (observe el recorrido del talón afectado).	1 = Ninguna.	El pie afectado no se abduce más que el pie del lado no afectado durante la oscilación.
	2 = Rotación moderada.	El pie afectado se abduce hasta el ancho de un zapato durante la oscilación.
	3 = Marcada.	El pie afectado se abduce más del ancho de un pie durante la oscilación.
10. Elevación de la cadera durante la oscilación media.	1 = Ninguna.	La pelvis desciende levemente durante la oscilación.
	2 = Elevación.	La pelvis se eleva durante la oscilación.
	3 = Salta.	Leve flexión de cadera. El sujeto contrae los músculos laterales del tronco y eleva la cadera durante la oscilación.
11. Flexión de rodilla desde el despegue del pie hasta la oscilación media *	1 = Normal.	La rodilla afectada se flexiona igual que la rodilla del lado no afectado.
	2 = Algo.	La rodilla afectada se flexiona, pero menos que la rodilla no afectada.
	3 = Mínimo.	Flexión mínima de la rodilla afectada (Casi no se observa la flexión)
	4 = Ninguna.	La rodilla permanece extendida durante la oscilación
12. Distancia de los dedos del pie al suelo.	1 = Normal	Los dedos del pie se separan del suelo durante a oscilación.
	2 = Ligero arrastre	Los dedos del pie se arrastran al principio de la fase de oscilación.
	3 = Marcado	Los dedos del pie se arrastran durante la mayor parte de la oscilación.
13. Rotación de la pelvis en la oscilación final.	1 = Hacia delante.	Rotación anterior de la pelvis para preparar el contacto de talon.
	2 = Neutra.	Postura erguida con la pelvis en rotación neutra.
	3 = Retraída.	La pelvis queda retrasada de manera marcada por detrás de la pelvis no afectada.
**Contacto de Talón Pierna Afectada**		
14. Contacto inicial del pie.	1 = Contacto de talon.	El talón hace contacto inicial con el suelo.
	2 = Apoyo con toda la planta.	El pie contacta con el peso distribuido en todo el pie.
	3 = No hay contacto de talon.	El pie contacta en el borde externo del pie o de los dedos.

* Los ítems 1 y 11 son baremados con 3/5 y 3/4 respectivamente, antes de añadir los ítems individuales a la puntuación total. Puntuación total = Suma (puntuación ítems 2–10 y 12–14) + (3/5 * (puntos ítem 1)) + (3/4 * (puntos ítem 11)). Puntuación mínima: 13.35. Puntuación máxima: 42. Una puntuación alta indica mayor afectación de la marcha.

## Data Availability

Not applicable.
